# Towards the design of epitope candidates for Coronavirus 2

**DOI:** 10.6026/97320630016375

**Published:** 2020-05-31

**Authors:** Hasanain Abdulhameed Odhar, Salam Waheed Ahjel, Suhad Sami Humadi

**Affiliations:** 1 Department of pharmacy, Al-Zahrawi University College, Karbala, Iraq

**Keywords:** SARS-CoV-2, 2019-nCoV, COVID-19, vaccine, epitope

## Abstract

The severe acute respiratory syndrome coronavirus-2, formerly known as 2019 novel coronavirus, is a pandemic public health threat. This beta coronavirus potentially infects the alveolar cells of the lung leading to pneumonia. The disease may progress into acute respiratory distress syndrome especially in elderly patients with comorbidities. Therefore, it is of interest to design and develop candidates for treatment, therapy and prevention. The spike glycoprotein of the virus known to potentially interact with angiotensin converting enzyme 2 as a cell entry receptor is a suitable candidate for further consideration as vaccine and treatment candidate. Hence, we screened the spike protein of coronavirus-2 for potential B-cell and T-cell epitopes for further deliberation. Thus, we document several peptides on the spike protein with predicted high antigenicity, low allergenicity and good stability against selected proteases. The linear B-cell epitope with sequence 'GFNCYFPLQSYGF' is of particular interest in this context towards the design and development of short peptide vaccine candidates for combat and care against the virus.

## Background

**Figure 1:**
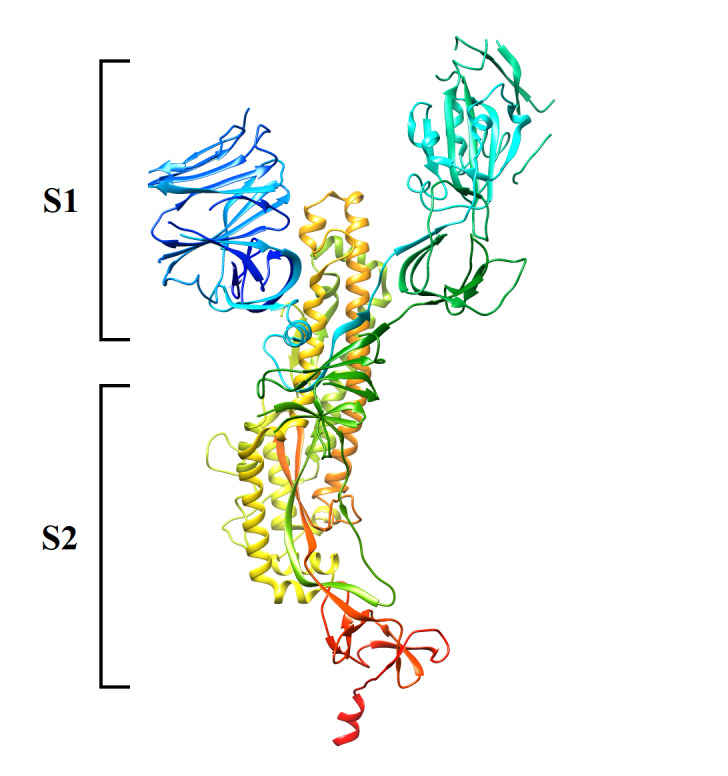
A three-dimensional cartoon illustration for chain A of SARS-CoV-2 spike protein, we have used spike protein crystal with code (6VXX) to generate this picture [16, 18]. The C-terminus is colored by red while N-terminus is colored by blue. As can be seen, the position of S1 and S2 subunits has been marked. UCSF chimera version 1.13.1 has been used to generate this three-dimensional representation [26].

In late December 2019, multiple cases of viral pneumonia were reported near a local seafood market in the Chinese city of Wuhan [1]. Based on genomic analysis results, a novel coronavirus was isolated from lower respiratory tract specimens of these patients. The newly identified RNA virus was provisionally named as 2019 novel coronavirus (2019-nCoV), but now is known as severe acute respiratory syndrome coronavirus-2 (SARS-CoV-2) [2,3]. This virus shares a genomic sequence identity of about 79% with another previously known beta coronavirus named as severe acute respiratory syndrome coronavirus (SARS-CoV) [4]. Coronavirus disease 2019 (COVID-19) patients are most commonly presented with fever, dry cough, fatigue and myalgia. Few patients may also show signs of rhinorrhea and diarrhea. Some COVID-19 cases are characterized by dyspnea which can progress in one week into acute respiratory distress syndrome (ARDS) [5,6]. It seems that elderly patients with comorbidities are at higher risk of developing severe illness [2,5, 7]. On March 11, 2020, the World Health Organization (WHO) had categorized COVID-19 outbreak as a pandemic threat [8]. According to COVID-19 situation report issued by WHO on April 08, 2020, the global confirmed cases are 1,353,361 and deaths are 79,235 [9]. The main route for SARS-CoV-2 transmission is through respiratory droplets as the virus can survive for about 2 hours in the air. However, digestive system and ocular surface may be also potential routes of viral transmission [10]. The mean incubation period for COVID-19 is estimated to be 6.4 days with a range of 2.1 to 11.1 days [11]. The basic reproduction rate R0 for COVID-19 outbreak is estimated to be 2.68, this necessitate the implementation of effective control measures [12]. Unfortunately, no drug or vaccine is currently approved to combat the outbreak of SARS-CoV-2 [13]. However, there are currently an accelerated efforts to repurpose some FDA approved drugs against COVID-19 like Chloroquine and Remdesivir [14]. Multiple programs are currently ongoing to develop SARS-CoV-2 vaccine by using whole virus, viral spike protein or mRNA [15]. SARS-CoV-2 can infect alveolar cells by using angiotensin-converting enzyme 2 (ACE2) as an entry receptor. The receptor-binding domain (RBD) is located within S1 subunit of the viral spike glycoprotein (S protein). The S1 subunit of SARS-CoV-2 spike protein shares about 70% identity with that of SARS-CoV. The spike protein of SARS-CoV-2 also contains S2 subunit that is responsible for viral membrane fusion with host cell. The S2 subunit of SARS-CoV-2 spike protein is highly conserved and shares about 99% identity with SARS-CoV [4,10]. The spike glycoprotein has been identified as a potential molecular target to develop a drug or vaccine that can interfere with viral entry process [15]. The spike protein has been isolated as a homo-trimeric crystal [16]. For simplicity of illustration, we will focus only on chain A of SARS-CoV-2 spike protein. A three-dimensional cartoon representation for this monomer can be seen in (Figure 1). In this study, we have evaluated chain A of SARS-CoV-2 spike protein with different immuno-informatics tools hosted by the Immune Epitope Database (IEDB) [17]. Our aim for this study is to predict epitopes for both T-cells and B-cells, these epitopes may facilitate future development of vaccine against SARS-CoV-2.

## Methodology:

### Setting up a screening plane:

**Figure 2:**
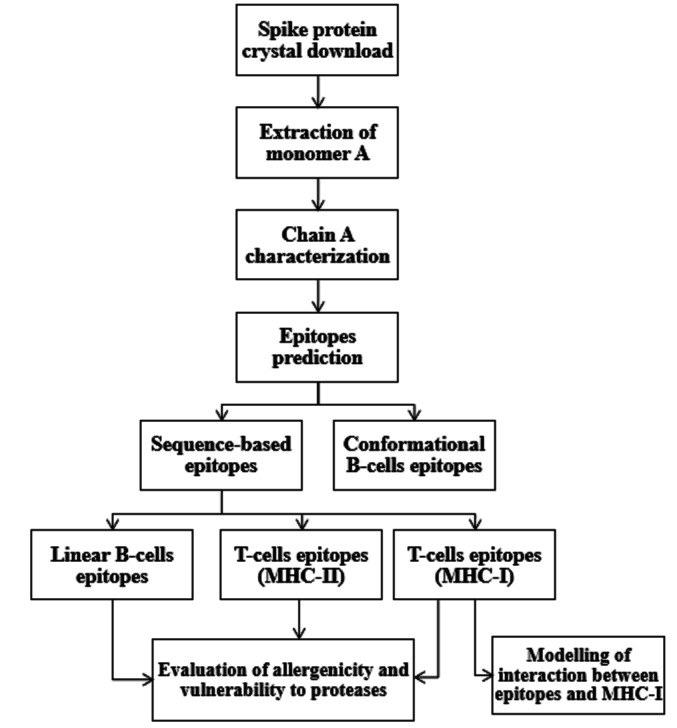
A flowchart representation of prediction pathway used for SARS-CoV-2 spike protein screening.

A concise overview for prediction pathway of this screening study can be summarized in a flowchart as seen in Figure 2.

### Characterization of physicochemical properties for chain A of SARS-CoV-2 spike protein:

The amino acids sequence of SARS-CoV-2 spike protein crystal with code (6VXX) was downloaded as FASTA file from protein data bank [16, 18]. For later use, the sequence of chain A was extracted from FASTA file in one letter format. We have used ProtParam tool to calculate different physicochemical properties for chain A residues sequence like molecular weight, isoelectric point, instability index and charge of the residues [19].

### Prediction of linear B-cells epitopes on chain A of SARS-CoV-2 spike protein:

The continuous B-cells epitopes were predicted by using antigen sequence properties tool provided by IEDB website [17]. The one letter sequence for only chain A of SARS-CoV-2 spike protein was submitted to the tool. Here, we have employed three epitopes prediction methods and these are: BepiPred-2.0 [20], Emini surface accessibility scale [21], Kolaskar and Tongaonkar antigenicity scale [22]. Regarding BepiPred-2.0, this prediction method depends on random forest algorithm that had been trained on epitopes of known antigen-antibody complexes [20]. The default threshold of 0.5 was used for interpreting BepiPred-2.0 results. On the other hand, Emini surface accessibility scale is based on prediction of surface probability by using specific formula [21]. Again, the default threshold value of 1.0 was used to evaluate this tool results. For Kolaskar and Tongaonkar antigenicity scale, this semi-empirical tool can predict peptide antigenicity by using physicochemical properties of residues and their frequencies in known B-cells epitopes [22]. For Kolaskar and Tongaonkar antigenicity scale output, the default threshold of 1.037 was used. To further refine the output of these three predictive methods, we have also calculated the antigenicity score for each predicted B-cells epitope by using VaxiJen v2.0 predictive tool. This tool can predict protective antigens by using physicochemical properties of submitted peptide with no need for alignment of residues sequence [23]. We have used a threshold value of >0.5 to predict antigenicity score. For linear B-cells epitopes prediction, we have only reported those peptides with antigenicity score greater than 0.5. Finally, the residues of these predicted epitopes were highlighted on chain A of the spike protein crystal as spheres with element based color by using PyMOL version 2.3 [24].

### Prediction of conformational B-cells epitopes on chain A of SARS-CoV-2 spike protein:

For the prediction of discontinuous B-cells epitopes, we have used DiscoTope version 2.0 tool which can be accessed through IEDB website [17]. This structure-based tool can predict B-cells epitopes by compiling data about solvent accessibility area, statistics of amino acid residues and spatial information [25]. For using this tool, we have downloaded SARS-CoV-2 spike protein crystal with code (6VXX) from protein data bank as PDB file [16,18]. Then, only chain A of the spike protein trimer was extracted by using UCSF chimera version 1.13.1 [26]. This extracted chain was submitted later to DiscoTope tool, we have used a threshold value of -2.5, which reflects a specificity of 80%. Finally, we have used PyMOL version 2.3 to mark the residues of these conformational epitopes on chain A of the spike protein crystal as spheres colored by element [24].

### Prediction of T-cells epitopes on chain A of SARS-CoV-2 spike protein as presented through major histocompatibility complex class I (MHC-I):

To predict peptides with intrinsic potential of being T-cells epitopes presented by MHC-I molecules, we have used a combined predictor tool available in IEDB website. This predictor tool can generate a final score for the affinity of the peptide towards proteasomal degradation, transporter associated with antigen processing (TAP), and also MHC-I molecules binding [17]. We have submitted chain A sequence as FASTA format with one letter code. This combined predictor tool offers the application of several different prediction methods to process data. We have selected NetMHCpan-2.0 method, this method had been trained on a large set of quantitative data for MHC binding with broad allelic coverage [27]. By using the most frequent alleles of human leukocyte antigen (HLA), we have screened the residues of chain A against 108 HLA alleles [28,29]. The length of the generated epitopes was set to 9 residues only as the MHC-I binding cleft seems to be tight and can present only short peptides [30]. Immuno-proteasome type of cleavage prediction has been also selected. We have reported only those peptides with VaxiJen antigenicity score greater than 0.5 [23].

### Prediction of T-cells epitopes on chain A of SARS-CoV-2 spike protein as presented through major histocompatibility complex class II (MHC-II):

We have used MHC-II binding prediction tool, available in IEDB epitope analysis resource, to scan chain A sequence for any peptide that can be presented by MHC-II molecules [17]. For this tool, we have used NetMHCIIpan-3.2 method to quantitatively anticipate the binding affinity of potential epitopes with MHC-II molecules. NetMHCIIpan-3.2 had been trained on extended data set obtained from IEDB site [31]. The sequence of chain A was submitted as FASATA format, and it has been screened against 27 HLA alleles that cover most of the population [28,29]. The length of the predicted peptides has been limited between 12 and 18 residues; this can cover about 82.89% of epitope frequency. The antigenicity score was predicted for output peptides by using VaxiJen v2.0 tool [23]. Only MHC-II binding peptides with antigenicity score greater than 0.5 have been reported.

### Prediction of allergenicity potential and stability against digesting enzymes for sequence-based epitopes:

To evaluate the stability of sequence-based epitopes, we have used PeptideCutter tool to predict vulnerability of these peptides against a number of proteases [32]. The sequence for each peptide was submitted as one letter code. For simplicity of presentation, we have only assessed the proteolytic potential of Arg-C proteinase, Neutrophil elastase, Asp-N endopeptidase and Trypsin. We have also used AllergenFP v.1.0 tool to predict the possibility of allergic reaction induction by these peptides. The concept of this tool depends on Tanimoto coefficient of similarity, auto-cross covariance transformation and principle component analysis (PCA) [33]. Again, the sequence of predicted epitopes was submitted in one letter format.

### Molecular modelling of interaction between epitopes and MHC-I molecules:

We have used Docktope tool to study interaction pattern between peptides and MHC-I molecules [34]. The molecular modeling by this web-based tool involves energy minimization and two rounds of extensive Vina docking [35]. The submitted epitopes were 9-mer long, they have been modelled against HLA-A*02:01 allele. The generated complex was downloaded as PDB file, LigPlot+ v.1.4.5 was used to identify amino acids residues involved in molecular interaction [36]. Then, we have used AutoDock vina version 1.1.2 to estimate free energy of binding for each complex [37]. UCSF chimera version 1.13.1 provides a user friendly interface to access AutoDock vina and prepare both epitope and MHC-I [26]. However, the exhaustiveness of search has been reduced to only 3.0 as protein-protein docking is considered an intensive computational process especially on local machines. Finally, we have used PyMOL version 2.3 to process three-dimensional cartoon images of molecular modelling [24].

## Results and discussion:

**Figure 3:**
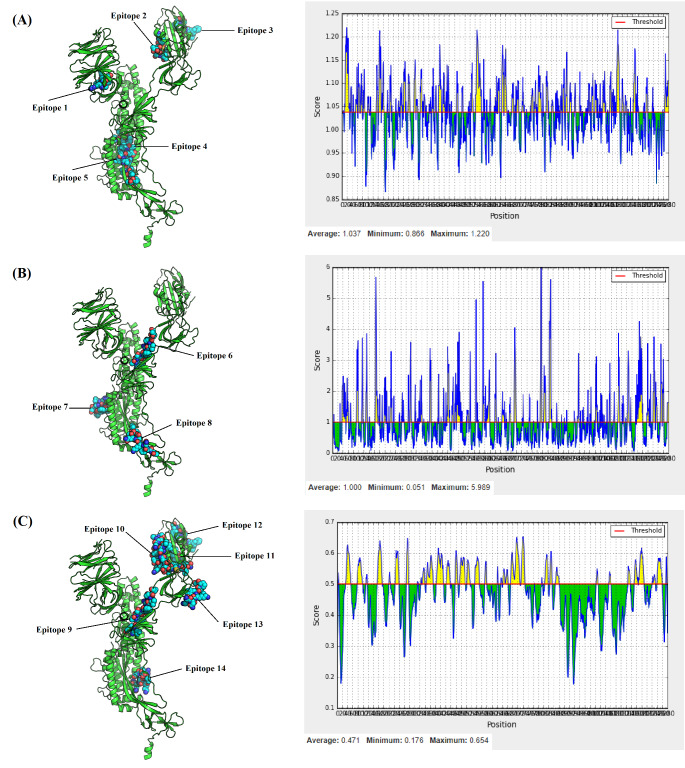
Position of linear B-cells epitopes along with their sequence-based scoring according to prediction method used. (A) Epitopes 1 to 5 were predicted by Kolaskar and Tongaonkar antigenicity method, (B) Epitopes 6 to 8 were predicted by Emini surface accessibility scale, and finally (C) Epitopes 9 to 14 were generated by using BepiPred-2.0.

**Figure 4:**
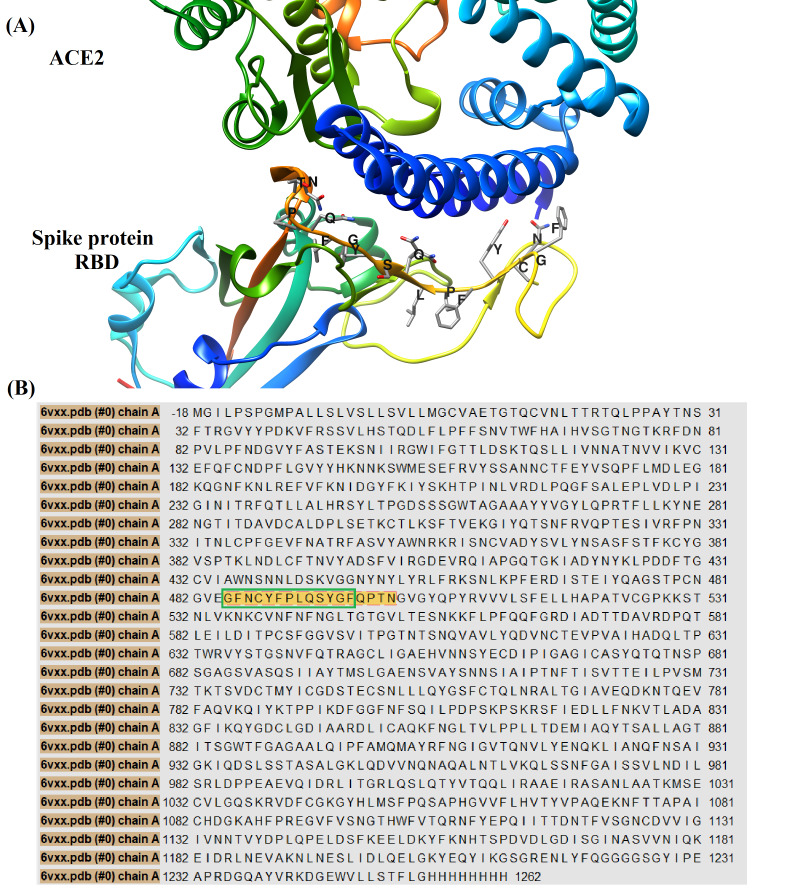
(A) A three-dimensional cartoon representation for interaction between receptor binding domain (RBD) of SARS-CoV-2 spike protein and angiotensin converting enzyme 2 (ACE2). The amino acid residues of spike protein RBD involved in interaction with ACE2 were labeled in one letter format, the sequence of these residues is (GFNCYFPLQSYGFQPTN). This figure was generated by using 6M0J crystal downloaded from protein data bank [41]. We have used UCSF chimera version 1.13.1 to process and render this image [26].(B) The amino acids sequence map for chain A of SARS-CoV-2 spike protein. The residues of spike protein involved in interaction with ACE2 were colored by gold within chain A sequence, the linear B-cells epitope with sequence (GFNCYFPLQSYGF) was encircled by green line. This sequence map was generated by using PDB crystal with code (6VXX) [16,18] and UCSF chimera version 1.13.1 [26].

**Figure 5:**
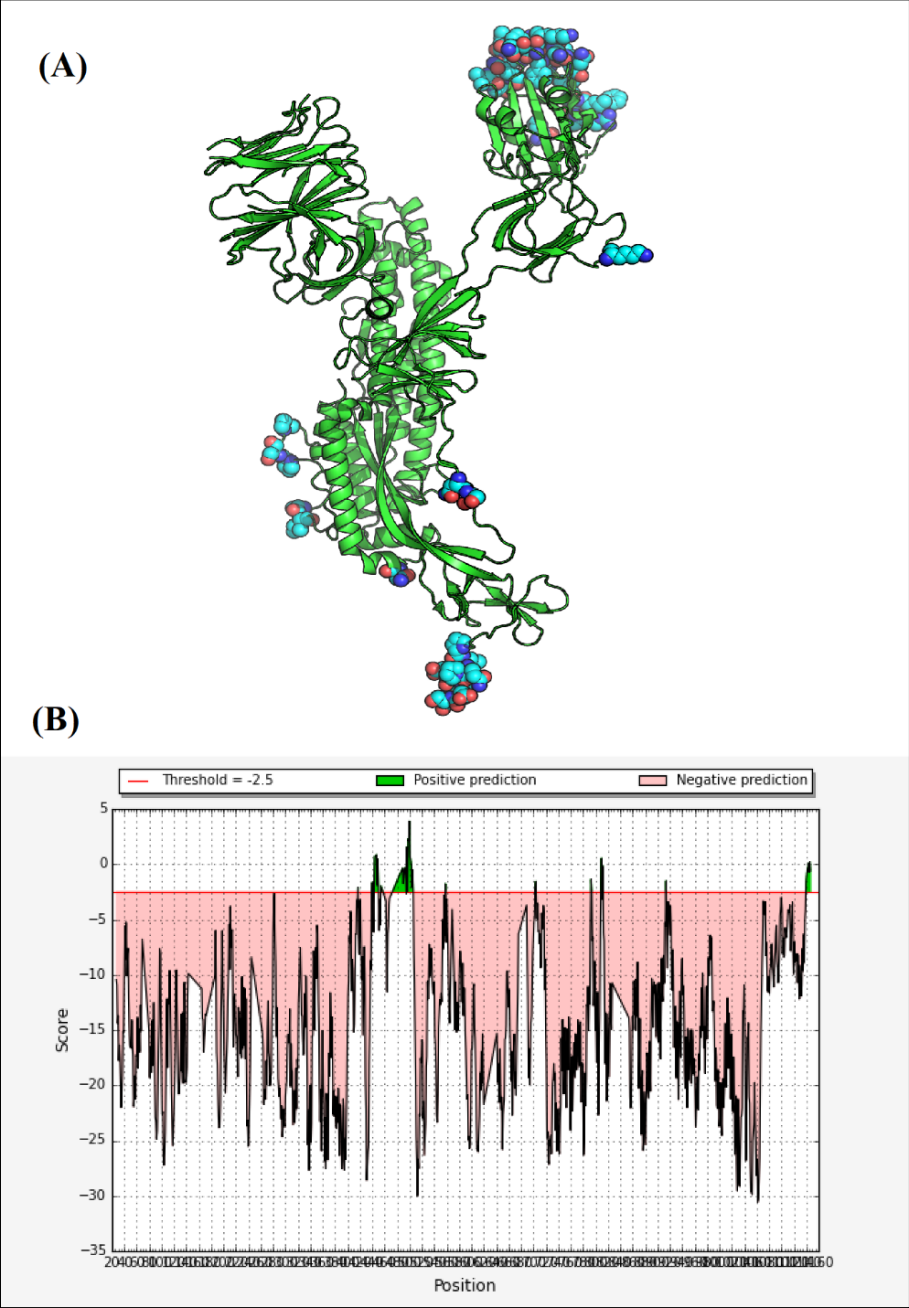
(A) Position of conformation B-cells epitopes on chain A of SARS-CoV-2 spike protein crystal. (B) Sequence-based scoring of these discontinuous epitopes as predicted by DiscoTope version 2.0 with a threshold red line of -2.5.

**Figure 6:**
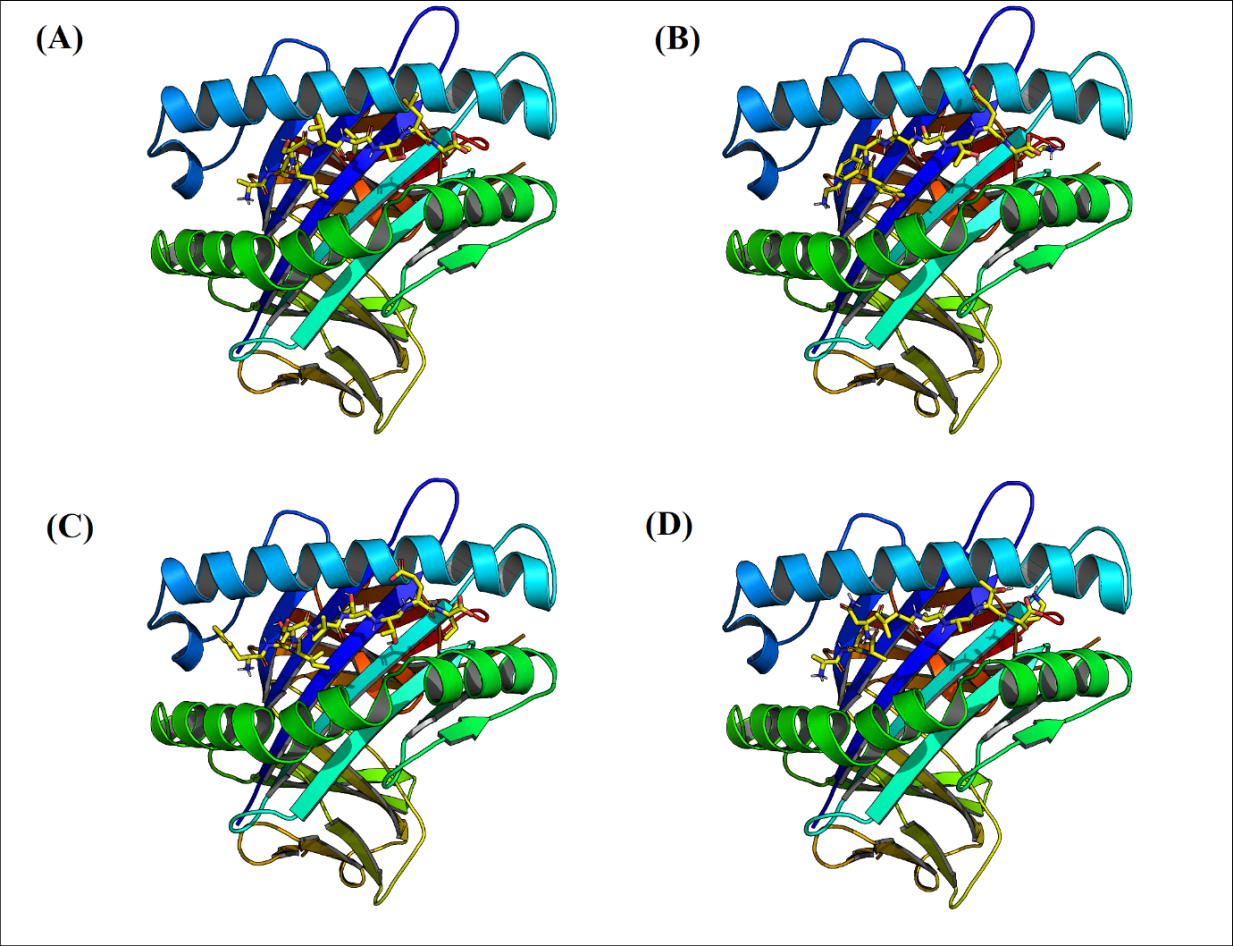
Three-dimensional cartoon illustration for molecular modeling of interaction between MHC class I molecule and T-cells epitopes with sequence: (A) ALLSLVSLL, (B) GVYFASTEK, (C) FTISVTTEI and (D) ASANLAATK.

The summary of physicochemical properties in (Table 1) indicates that chain A of spike protein has a net negative charge as the isoelectric point (PI) < 7. It is well-known that isoelectric point is the solution PH at which the protein has a neutral charge [38]. Also, the total number of negatively charged residues is greater than those with positive charge. The calculated instability index (II) for chain A is 31.26; proteins with instability index value less than 40 are usually stable [39]. Fourteen linear B-cells epitopes were predicted according to the submitted sequence of chain A for spike protein. The corresponding position, length, sequence and antigenicity for each peptide can be seen in (Table 2). According to prediction method employed, the position of these continuous epitopes along with their sequence-based scoring is shown in (Figure 3). Most of these predicted epitopes are located in solvent accessible regions within chain A crystal, this may facilitate recognition by membrane bound immunoglobulins of B-cells. Of interest is the linear epitope number 12, this epitope seems to be part of the receptor binding domain (RBD) in SARS-CoV-2 spike protein that is involved in interaction with the entry receptor ACE2 [40]. The sequence of this linear epitope can be recognized in (Figure 4), which shows spike protein RBD residues involved in interaction with ACE2. According to (Table 3), forty-two residues were identified as B-cells conformational epitopes on chain A of SARS-CoV-2 spike protein. It is worth noting that conformational epitope residues from Tyrosine 489 to Asparagine 501 are believed to be also part of spike protein receptor binding domain as can be seen in (Figure 4) [40]. In this table, the contacts number variable refers to number of Cα atoms within 10-angstrom distance from Cα atom of a particular residue. A residue with a low contacts number value is usually located near antigen surface. On the other hand, the propensity score measures the probability of being part of a conformation epitope for a particular amino acid residue. Finally, the DiscoTope score is calculated by combining both propensity score and contacts number for each residue. DiscoTope scores above the threshold line represent positive epitope predictions (green area) while negative predictions (orange area) are usually generated with DiscoTope scores below the threshold [17,25]. The position of these discontinuous epitopes with their sequence-based scoring can be seen in (Figure 5). The epitopes for T-cells presented through MHC-I molecules can be seen in (Table 4). These seven epitopes were ranked according to their location within chain A sequence. The score values for proteasomal cleavage, TAP mediated transport and MHC-I binding reflect efficiency of processing by these three consecutive elements. Efficient presentation by MHC-I pathway is usually associated with higher score values [17]. Regarding MHC-II presentation pathway, only four T-cell epitopes with high antigenicity score were predicted. As can be seen in (Table 5), these epitopes have different length between 14-mer and 18-mer. According to IEDB guide, peptides with low adjusted rank value are usually good binder to MHC class II [17]. Evaluation of stability against digesting enzymes and allergenicity potential for sequence-based epitopes can be seen in (Table 6). Unfortunately, many of linear B-cells epitopes are predicted to be allergenic with variable degree of susceptibility to enzymatic degradation. Of interest is the continuous B-cells epitope number 12, this peptide appears to be a stable candidate due to its predicted resistance for degradation by the selected four proteases. This epitope is also expected to be non-allergenic. Most of T-cells epitopes are probably non-allergenic with good stability against the selected proteases. Finally, molecular modeling results showed that four of the predicted T-cells epitopes have similar interaction pattern with MHC-I molecules. Based on (Table 7), these epitopes have a comparable free energy of binding. Examining three-dimensional images of interaction, as seen in (Figure 6), suggest that these peptides can exhibit a minimal energy pose situated well within MHC-I binding groove.

## Conclusions:

We document the linear B-cell epitope 'GFNCYFPLQSYGF' of specific relevance towards the design and development of short peptide vaccine candidates for combat and care against coronavirus-2.

## Figures and Tables

**Table 1:** Physicochemical properties calculated for chain A of SARS-CoV-2 spike protein.

Property	Computed value
Number of residues	1281
Molecular weight	141410.94 kDa
Isoelectric point (PI)	6.09
Negatively charged residues	111
Positively charged residues	99
Instability index	31.26

**Table 2:** Linear B-cells epitopes predicted on chain A for SARS-CoV-2 spike protein.

No.	Position	Length	Sequence	Antigenicity	Prediction method
1	53	8	RGVYYPDK	1.019	Kolaskar and
2	395	10	TFKCYGVSPT	1.506	Tongaonkar antigenicity
3	507	8	CYFPLQSY	0.939	
4	742	8	TTEILPVS	1.207	
5	1076	14	PHGVVFLHVTYVPA	0.806	
6	333	10	QTSNFRVQPT	1.405	Emini
7	827	10	DPSKPSKRSF	0.815	surface accessibility
8	1087	9	VPAQEKNFT	1.011	
9	331	11	IYQTSNFRVQP	1.015	BepiPred-2.0
10	385	29	SVLYNSASFSTFKCYG VSPTKLNDLCFTN	1.132	
11	424	16	DEVRQIAPGQTGKIAD	1.039	
12	504	13	GFNCYFPLQSYGF	0.852	
13	574	8	SNKKFLPF	1.395	
14	1054	9	GQSKRVDFC	1.779	

**Table 3:** Conformational epitopes for B-cells predicted on chain A of SARS-CoV-2 spike protein.

No.	Position	Residue name	Contacts number	Propensity score	DiscoTope score
1	417	LYS	12	-0.813	-2.099
2	440	ASN	5	-1.222	-1.657
3	443	SER	19	0.432	-1.803
4	444	LYS	8	1.79	0.664
5	447	GLY	14	2.779	0.849
6	448	ASN	27	1.336	-1.922
7	449	TYR	3	0.877	0.431
8	450	ASN	15	-0.334	-2.021
9	454	ARG	14	-0.401	-1.965
10	489	TYR	0	-0.418	-0.37
11	490	PHE	9	-0.819	-1.76
12	491	PRO	11	0.123	-1.156
13	492	LEU	11	1.006	-0.375
14	493	GLN	11	1.042	-0.343
15	494	SER	14	0.753	-0.944
16	496	GLY	2	2.019	1.557
17	497	PHE	27	0.723	-2.465
18	498	GLN	4	3.076	2.262
19	499	PRO	4	2.854	2.066
20	500	THR	1	4.501	3.868
21	501	ASN	24	3.882	0.676
22	503	VAL	2	0.217	-0.038
23	504	GLY	3	-2.009	-2.123
24	505	TYR	10	0.727	-0.507
25	506	GLN	14	-0.912	-2.417
26	558	LYS	0	-2.012	-1.781
27	703	ASN	3	-2.23	-2.318
28	704	SER	3	-1.384	-1.57
29	793	PRO	0	-1.559	-1.38
30	794	ILE	4	-2.173	-2.383
31	809	PRO	5	-1.511	-1.912
32	810	SER	4	1.115	0.526
33	812	PRO	3	0.186	-0.18
34	914	ASN	7	-0.803	-1.516
35	1140	PRO	8	-0.752	-1.586
36	1141	LEU	3	-0.71	-0.974
37	1142	GLN	7	0.439	-0.416
38	1143	PRO	6	0.445	-0.296
39	1144	GLU	4	0.586	0.059
40	1145	LEU	5	-0.203	-0.754
41	1146	ASP	6	1.013	0.206
42	1147	SER	5	-0.017	-0.59

**Table 4:** Prediction of T-cells epitopes presented through MHC-I binding pathway.

No.	Position	Length	Peptide	Proteasome score	TAP score	MHC-I score	Antigenicity Score
1	11	9	ALLSLVSLL	1.38	0.43	-1.27	0.649
2	108	9	GVYFASTEK	0.89	0.21	-1.11	0.711
3	208	9	GAAAYYVGY	1.24	1.29	-2.67	0.66
4	436	9	KIADYNYKL	1.68	0.51	-1.2	1.664
5	737	9	FTISVTTEI	0.93	0.27	-0.85	0.854
6	1039	9	ASANLAATK	1	0.35	-1.1	0.701
7	1079	9	VVFLHVTYV	0.88	0.26	-1.03	1.512
MHC: major histocompatibility complex; TAP: Transporter associated with antigen processing.

**Table 5:** Prediction of T-cells epitopes presented through MHC-II binding pathway.

No.	Position	Length	Peptide	Adjusted rank	Antigenicity score
1	249	18	PIGINITRFQTLLALHRS	0.33	0.78
2	530	14	VVLSFELLHAPATV	0.31	0.865
3	905	15	WTFGAGAALQIPFAM	0.32	0.667
4	710	16	SIIAYTMSLGAENSVA	0.25	0.574

**Table 6:** Vulnerability to digesting enzymes and allergenicity potential of predicted epitopes

No.	Epitope	Vulnerability to digesting enzymes				Allergenicity
		Arg-C proteinase	Neutrophil elastase	Asp-N endopeptidase	Trypsin	
Linear B-cells epitopes.						
1	RGVYYPDK	Yes	Yes	Yes	Yes	No
2	TFKCYGVSPT	No	Yes	No	Yes	Yes
3	CYFPLQSY	No	No	No	No	Yes
4	TTEILPVS	No	Yes	No	No	Yes
5	PHGVVFLHVTYVPA	No	Yes	No	No	Yes
6	QTSNFRVQPT	Yes	Yes	No	Yes	Yes
7	DPSKPSKRSF	Yes	No	No	Yes	Yes
8	VPAQEKNFT	No	Yes	No	Yes	Yes
9	IYQTSNFRVQP	Yes	Yes	No	Yes	Yes
10	SVLYNSASFSTFKCYG	No	Yes	Yes	Yes	No
	VSPTKLNDLCFTN					
11	DEVRQIAPGQTGKIAD	Yes	Yes	Yes	Yes	No
12	GFNCYFPLQSYGF	No	No	No	No	No
13	SNKKFLPF	No	No	No	Yes	Yes
14	GQSKRVDFC	Yes	Yes	Yes	Yes	Yes
T-cells epitopes (MHC-I).						
1	ALLSLVSLL	No	Yes	No	No	No
2	GVYFASTEK	No	Yes	No	Yes	No
3	GAAAYYVGY	No	Yes	No	No	No
4	KIADYNYKL	No	Yes	Yes	Yes	Yes
5	FTISVTTEI	No	Yes	No	No	No
6	ASANLAATK	No	Yes	No	Yes	No
7	VVFLHVTYV	No	Yes	No	No	Yes
T-cells epitopes (MHC-II).						
1	PIGINITRFQTLLALHRS	Yes	Yes	No	Yes	No
2	VVLSFELLHAPATV	No	Yes	No	No	No
3	WTFGAGAALQIPFAM	No	Yes	No	No	No
4	SIIAYTMSLGAENSVA	No	Yes	No	No	No

**Table 7:** Molecular docking results for interaction between predicted T-cells epitopes and MHC-I binding molecules.

No.	Epitope	Binding energy (Kcal/mol)	MHC-I Interacting residues
1	ALLSLVSLL	-7.1	Tyr07, Glu63, Lys66, Asp77, Tyr99, Lys146, Trp147, Tyr171
2	GVYFASTEK	-7	Glu63, Lys66, Thr73, Asp77, Tyr99, Tyr123, Thr143, Lys146, Trp147
3	FTISVTTEI	-7	Tyr07, Glu63, Lys66, Thr73, Asp77, Tyr99, Lys146, Trp147, Tyr171
4	ASANLAATK	-6.2	Glu63, Arg65, Lys66, Thr73, Asp77, Tyr99, Trp147, Tyr171
